# *Aloe arborescens*: In Vitro Screening of Genotoxicity, Effective Inhibition of Enzyme Characteristics for Disease Etiology, and Microbiological Activity

**DOI:** 10.3390/molecules27072323

**Published:** 2022-04-03

**Authors:** Kamil Pawłowicz, Szymon Sip, Tomasz Plech, Barbara Kaproń, Joanna Kobus-Cisowska, Judyta Cielecka-Piontek

**Affiliations:** 1 Phytopharm Klęka S.A., Klęka 1, 63-040 Nowe Miasto nad Warta, Poland; kamil.pawlowicz@europlant-group.pl; 2 Department of Pharmacognosy, Poznan University of Medical Sciences, Rokietnicka 3, 60-806 Poznan, Poland; szymonsip@ump.edu.pl; 3 Department of Pharmacology, Medical University of Lublin, Chodźki 4a, 20-093 Lublin, Poland; tomasz.plech@umlub.pl; 4 Department of Clinical Genetics, Medical University of Lublin, Radziwiłłowska 11, 20-080 Lublin, Poland; barbara.kapron@umlub.pl; 5 Department of Gastronomy Sciences and Functional Foods, Faculty of Food Science and Nutrition, Poznan University of Life Sciences (PULS), Wojska Polskiego Str. 28, 60-637 Poznan, Poland; joanna.kobus-cisowska@up.poznan.pl

**Keywords:** *Aloe arborescens*, in vitro safety, enzyme inhibitions, scratch test, microbiological activity

## Abstract

The present study assessed the genotoxicity, the possibility of inhibiting selected enzymes, and the microbial activity of lyophilisate from 3-year-old *A. arborescens* leaves obtained from controlled crops. The lyophilisate from 3-year-old *A. arborescens* leaves was standardized for aloin A and aloenin A content. Moreover, concentrations of polyphenolic compounds and phenolic acids were determined. The first stage of the research was to determine genotoxicity using the comet test, which confirmed the safety of *A. arborescens*. Assays of enzymatic inhibition were performed for hyaluronidase (IC50 = 713.24 ± 41.79 µg/mL), α-glucosidase (IC50 = 598.35 ± 12.58 µg/mL), acetylcholinesterase and butyrylcholinesterase (1.16 vs. 0.34 µM of eserine/g d.m., respectively). The next stage of the research was to determine the ability of the healing properties using the scratch test, which showed a positive response using the extract. Microbial activity was evaluated and obtained against Gram-negative and Gram-positive bacteria and yeasts. We concluded that *A. arborescens* leaf gel meets the important conditions for plant raw materials to obtain semi-solid forms of herbal medicinal products.

## 1. Introduction

Active substances in plants have been of interest to humankind for centuries. From the earliest recorded times, plants were both food and an essential principle of medicine. This trend has survived to the present day. Even though synthetic drugs lead the way in many therapies, herbal medicine, which is often the foundation for developing synthetic drugs, is still present in prophylaxis, primary, and adjuvant therapy.

Natural compounds exhibit biological activity due to affinity for specific enzymes or receptors through structural similarity to the substrates of physiological processes [1,2]. Plant-derived chemicals may play the role of agonists/antagonists of many systems that determine the organism’s homeostasis (e.g., atropine, reserpine, caffeine) [3,4].

Secondary metabolites, which are not directly related to plant growth and development, are responsible for the pharmacological activity of plants. Due to the plentiful compounds in raw plant materials, it is often difficult to identify the specific compounds responsible for the action. The synergism of compounds coexisting in extracts allows for a multitarget approach to eliminating specific diseases. For example, the inhibition of selected enzymes may be associated with the neutralization of the free radicals in the inflammation process, often resulting from the synergistic action of compounds present in plant extracts [5,6,7]. However, the issue is complex, as individual compounds may become the basis for synthetic drugs, so one form has no clear advantage.

Initially, the therapeutic goal was to obtain the required concentration of active substances at the site of the planned action [8]. Bearing in mind the possibility of the interaction of extracts of active compounds with many therapeutic goals, and the need to achieve the concentration of the active compound at the site of action, we conducted screening tests of the biological activity of the lyophilisate obtained from the *Aloe arborescens* pulp. 

The *Aloe* genus has been known for centuries for its multidirectional uses. It includes over 500 species, which may differ significantly in external appearance, from lianas to woody forms [9]. The common element for all species is leaves with pulp; this is where the richness of the compounds is contained. *Aloe* gel can have a water content of up to 99.5%; despite this, the remaining mass, although small, attracts the attention of researchers. Over 75 substances have already been indicated as potentially biologically active [10]. These include, but are not limited to, polysaccharides, phenolic compounds, minerals, and vitamins soluble in fat and water [11]. Therefore, *Aloe* has been, and still is, used in medicine—its wide range of active compounds can be intended for external, topical application, or ingestion.

Among the *Aloe* genus, the most famous species is *Aloe vera*, and numerous studies confirm its activity [12,13,14,15,16]. For *A. arborescens*, the number of reports is not that great; however, the medicinal properties of the species are beginning to be noticed and appreciated. The studies conducted so far consider *A. arborescens* as a genus with therapeutic effects [17,18,19]. Antitumor activity has been demonstrated, for example, against cancers of the intestine, rectum, duodenum, liver, pancreas, and skin [20]. In addition, alcoholic extract from *A. arborescens* leaves exhibits high activity against enzymes such as α-amylase and peroxidase [21]. The literature on the subject also indicates the high potential of the microbiological activity of the raw material in its broad spectrum of activity [22]. Thus, *A. arborescens*, despite being less popular than *Aloe vera*, shows significant biological activity and thus has high research potential to accurately determine its activity profile and the possibility of using it as a medicinal raw material. Previous research has investigated how the age of *A. arborescens* affects the content of aloin A, aloenin A, polyphenolic compounds, and phenolic acids [23]. 

Bearing in mind the multidirectional biological activity of extracts from various species of the *Aloe* genus and the lack of complex screening of biological activity for the species of *A. arborescens* in this paper, we have proposed research directions that will indicate the best pharmaceutical application for the tested plant material. Therefore, the present study involved in vitro studies aimed at assessing the potential of lyophilisate from 3-year-old *A. arborescens* leaves for the prevention and/or treatment of disorders caused by the disturbances in hyaluronidase α-glucosidase, acetyl- and butyrylcholinesterase activities. Moreover, combined results obtained from the antimicrobial activity evaluation, scratch, and comet assays were analyzed to check the investigated plant material’s genotoxicity and possible wound healing and antiseptic potential.

## 2. Results

This research is a continuation of our previous studies, which included the analysis of the content of aloin A, aloenin A, polyphenolic compounds, and phenolic acids in raw material, and the evaluation of the antioxidant activity of extracts from the freeze-dried leaf pulp of *A. arborescens* from 1-, 2-, 3- and 4-year-old plants. The effects of the study mentioned above can be found in one of our published papers [23]. This manuscript presents the results of our research on the lyophilizate from 3-year-old plants, which turned out to exhibit a higher activity and height content of active compounds (Table 1). 

**Figure 1:**
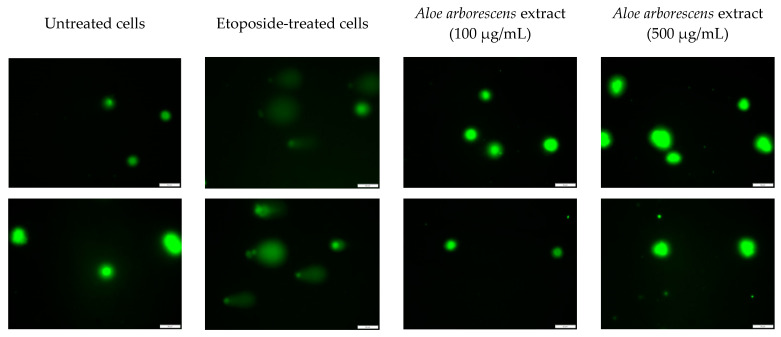
Genotoxicity evaluation of *A. arborescens* extract in Hs27 cells (human normal skin fibroblasts). The investigated extract was tested at 100 and 500 µg/mL. Etoposide (positive control) was tested at 10 µg/mL. Pictures were obtained using a fluorescence microscope showing the occurrence of the so-called tailing effect.

Firstly, the genotoxicity assessment was carried out by applying a comet assay using the human fibroblast Hs27 cell line, which revealed no genotoxic properties for two concentrations of the extracts from lyophilized *A. arborescens* gel (Figure 1). Etoposide, a known genotoxic agent, was used as a positive control, confirmed by a visible tailing effect corresponding with the genotoxic effect.

**Figure 2:**
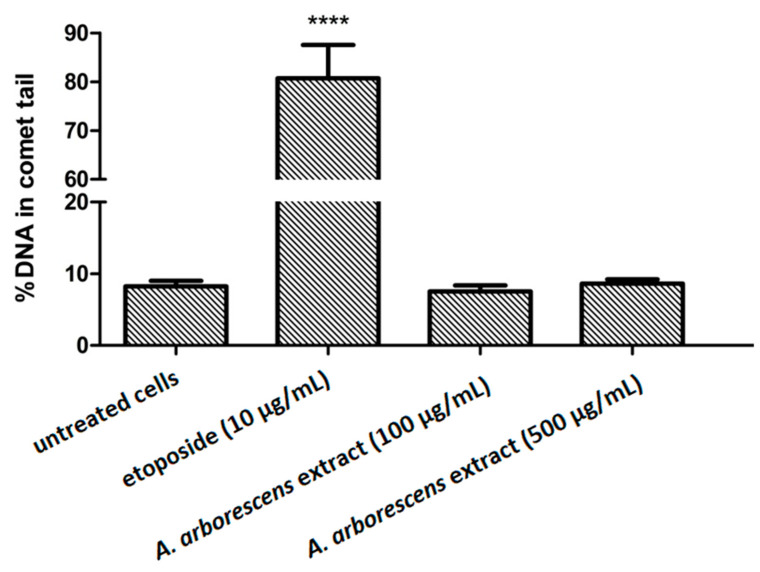
Quantitative analysis of the DNA damage in Hs27 cells after 24 h exposition to etoposide (positive control, 10 µg/mL) or *A. arborescens* extract. Data are shown as mean ± SEM. Statistical significance was calculated using ANOVA analysis followed by Dunnett’s post hoc test (**** *p* < 0.0001 when compared to untreated cells).

Statistical test carried out for the quantitative analysis of the DNA damage after treating with *A. arborescens* and etoposide allowed us to observe that the damage level for the extracts was comparable to the cells not treated with a genotoxic agent (<10% DNA in comet tail). In contrast, in the case of etoposide-treated cells, a statistically significant increase in damage was observed (>80% DNA in comet tail) (Figure 2). 

The activity of the tested extract from the freeze-dried gel of 3-year-old *A. arborescens* leaves against enzymes was carried out for hyaluronidase. Hyaluronic acid is an essential skin constituent, and plays a role in the wound healing process and in maintaining tissue hydration [24]. The inhibitory potential of hyaluronidase activity, the enzyme involved in the degradation of hyaluronic acid, was selected for the evaluation of anti-inflammatory properties of the studied extract as hyaluronic acid degradation products exhibit pro-inflammatory properties, and the high level of low molecular weight hyaluronic acid produced may be the result of excessive hyaluronidase activity [25]. In the conducted study, the IC50 value was determined for the extract from the freeze-dried pulp of 3-year-old *A. arborescens* leaves, which was 660.00 ± 38.52 µg/mL. This was more potent than the reference substance—kaempferol—for which the IC50 was 713.23 ± 41.79 µg/mL.

As one of the effects of the studied extract on the gastrointestinal tract, the antidiabetic potential was investigated. For this purpose, the possibility of inhibiting the activity of α-glucosidase was examined. α-Glucosidase inhibitors are drugs with an antihyperglycemic mechanism used to treat diabetes, especially type 2 diabetes. Firstly, the assay was performed for the reference standard—acarbose. These results were a reference and allowed the comparison with the IC50 determined for the extract. The tested extract inhibited the activity of α-glucosidase more potently than acarbose (IC50 values were 598.35 ± 12.58 µg/mL and 893.22 ± 33.07 µg/mL, respectively).

**Figure 3:**
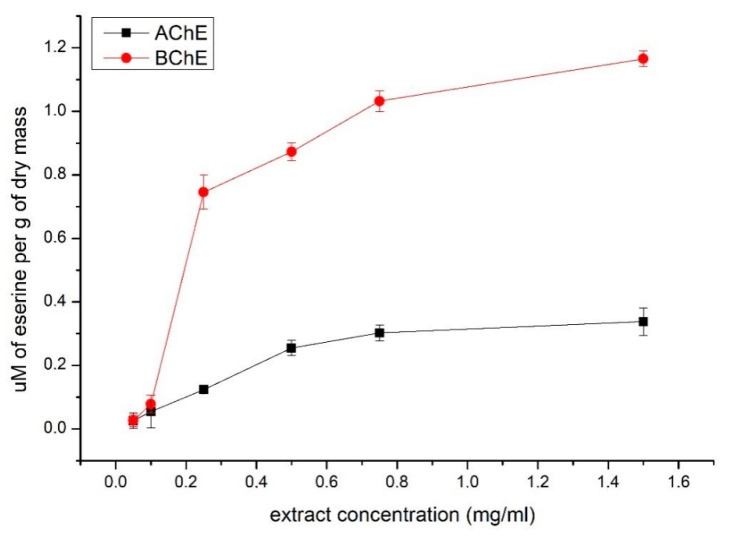
The percentage of AChE and BChE inhibition expressed as µM of eserine per gram of dry mass.

The conducted acetylcholinesterase (AChE) and butyrylcholinesterase (BChE) activity inhibition tests were performed to assess the neuroprotective potential of the 3-year-old *A. arborescens* lyophilisate extract. The study results showed that the extract inhibited BChE almost 3.5-fold more strongly than AChE (1.16 vs. 0.34 µM of eserine/g d.m., respectively) (Figure 3), thus showing some binding specificity to the enzymes tested.

**Figure 4:**
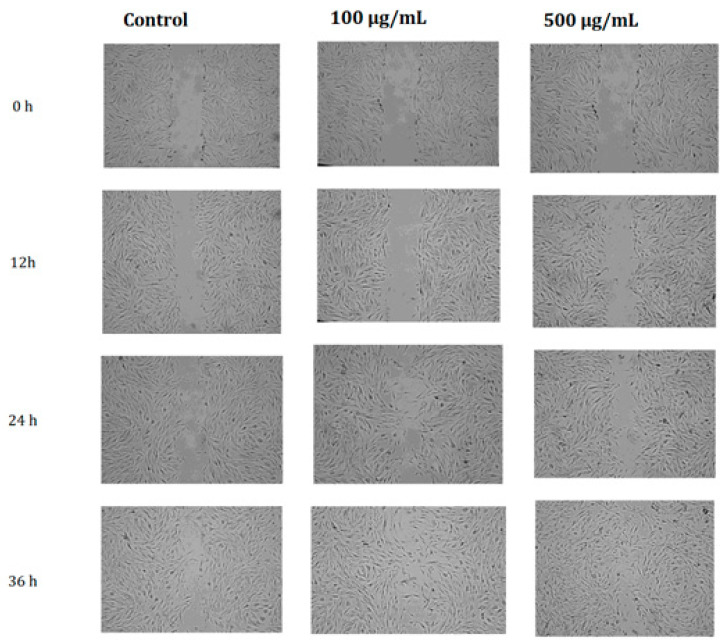
Microscopic photographs show scratch wound closure for control and selected extract concentrations at 0 h, 12 h, 24 h, and 36 h, with a visible disappearance of the scratch using the tested extract.

The wound healing scratch test was evaluated, given the possible application to the skin. Comparing the effect of *A. arborescens* lyophilisate extracts with the control sample on cell migration, which simulates the wound healing process, it was noted that after 36 h, the highest concentration of the extract showed the highest degree of atresia of the wound. At the same time, the lower concentration of the extract still gave a positive response compared to the control sample (Figure 4).

Another aspect tested was the antimicrobial activity. The study was carried out for selected strains of Gram-positive and Gram-negative bacteria, and yeast in the well diffusion test, which obtained the diameters of the inhibition zones, as presented in Table 2. For most selected strains, infection occurs mainly through the gastrointestinal tract and to a lesser extent through the respiratory tract or damaged skin.

The size of the inhibition zone was the largest for *Klebsiella pneumoniae* among Gram-negative bacteria (11.3 mm), while for Gram-positive bacteria, the most substantial growth inhibition by the extract was observed for *Staphylococcus auerus* (9.1 mm). *Clostridium* spp. and *Enterobacter aerogenes* were the least susceptible to the antimicrobial effect of the tested extract. The diameters of inhibition zones for yeasts (9.2 mm for *Candida krusei* and 10.7 mm for *Candida albicans*) were at the level between that obtained for Gram-positive and harmful bacteria. 

## 3. Discussion

Aloe species are plants that have been known for centuries and willingly used for their healing properties. Their laxative properties are the best known and, at the same time, the most studied, but they are also used in, e.g., wound healing or immunostimulation [26,27]. Considering the multitude of compounds found in various parts of these plants, a broad spectrum of activity can be expected, and many of the effects of Aloe compounds still need further experimentation.

Our previous research on the plant material *Aloe arborescens* showed that the lyophilisates obtained from the pulp of 3-year-old leaves contain the highest polyphenol content, which translates into the highest antioxidant activity against the DPPH radical [23]. Bearing in mind the results obtained earlier, we focused on assessing the safety and activity potential of lyophilisates from 3-year-old *Aloe arborescens* leaves in the current research.

From the literature on the subject, it is known that selected species of *Aloe* show antiproliferative [28,29] and immunostimulating [30] properties; therefore, the comet test was carried out to assess potential genotoxicity. The results obtained in vitro indicate the lack of genotoxic effects on human cells during the use of the lyophilisate from the gel of 3-year-old *A. arborescens* leaves on the skin, as the fibroblasts exposed to the studied extract in two concentrations did not show signs of DNA damage, and results were similar to cells not treated with a genotoxic agent (etoposide). The toxicity issue, including genotoxicity, of *Aloe* genus species, is complicated as the literature presents both for and against arguments [31,32,33,34]. Researchers assume plant genotoxicity often points to hydroxyanthracene derivatives (e.g., aloin, aloe-emodin) as compounds responsible for this effect [35]. An argument supporting this theory is that non-decolorized *Aloe vera*, without the reduced content of hydroxyanthracene derivatives, administered orally to mice and rats, caused intestinal lesions [36]. This effect was not observed when low anthraquinone *Aloe* preparations were used [37]. Moreover, the results obtained by Hu et al. showed that purified *Aloe vera* whole leaf dry juice showed no toxicity to the colon and kidney after oral administration [38]. The literature data are mainly related to *A. vera*, while information on *A. arborescens* is limited. A one-year study in rats found that orally administered whole leaf powder of *Aloe arborescens* introduced into the diet of animals at concentrations of 0.16%, 0.8%, and 4%, in addition to hematological changes, caused diarrhea, weight loss, dilatation in the lymph nodes, and pigmentation in the lymph nodes and renal tubule [39]. Considering the adverse effects observed after the administration of whole leaf or non-decolorized raw material, an attempt to use the pure leaf gel or its decolorized form seems promising, as anthranoid compounds are present in the leaf peel/latex [40].

When the safety of use in the test on fibroblasts was confirmed, screening studies were carried out, starting with testing the activity of the extract against selected enzymes.

The ability to inhibit the activity of hyaluronidase, which is involved in the inflammatory process, indicates the anti-inflammatory potential of the lyophilisate of *A. arborescens* leaf pulp. This is in line with literature reports that mention the anti-inflammatory effects of *Aloe* plants in various diseases [41,42,43]. Similar IC50 values were found for *Aloecamperi* and *Aloepercrassa* latex; Gebrelibanos et al. received an IC50 of 771.78 µg/mL and 664.47 µg/mL, respectively [44]. In our study, we used a freeze-dried *A. arborescens* gel, whose main ingredients were water and polysaccharides, while Gerbrelibanos et al. studied latex with significant anthracompounds. Considering the differences in the chemical composition of these two raw materials, one can conclude that the action of several groups of active compounds determines the effect of the hyaluronidase inhibition mechanism. 

The study confirmed that α-glucosidase activity was inhibited more strongly than acarbose, a drug used in antidiabetic therapy. The beneficial effects of administering *Aloe vera* to patients with impaired glycaemic levels are known in traditional folk medicine and have been confirmed in vitro and in vivo, and even in clinical trials [45,46]. These tests mentioned in the literature included various species, parts of plants, and preparation methods, which influenced the results, but the results indicated the effectiveness despite these differences. None of the studies known to the authors clearly stated the mechanism of antihyperglycemic action, but in many cases, researchers observed the ability to inhibit enzymes, including a-glucosidase [12,47,48,49].

Research also included assessing the neuroprotective potential of *A. arborescens* lyophilisate. AChE and BChE inhibition studies were chosen as models. Given the argued anti-inflammatory and antioxidative properties of the selected *Aloe* species, researchers are trying to investigate the protection of the nervous system, as these properties could decrease oxidative stress and inflammation, which are associated with many neurodegenerative diseases. These assumptions were confirmed in studies on rats where these effects proved helpful in treating ischemia, epilepsy, and Parkinson’s disease [50,51,52]. Despite the often-unknown proper protective mechanism of action, the anti-inflammatory and antioxidant properties increase the value of the raw material in terms of use in diseases related to neurodegeneration. After confirming the presence of antioxidant and anti-inflammatory properties for our raw material, we decided to continue a step further and check the ability of the tested raw material to inhibit the activity of AChE and BChE, enzymes responsible for the degradation of choline esters, including acetylcholine and butyrylcholine. We obtained an AChE and BChE inhibitory effect, and for BChE inhibition, this effect was more substantial. One of the theories to explain the pathogenesis of Alzheimer’s disease is the cholinergic hypothesis, on which current treatment standards are based [53]. Clementi et al. also tested 3-year-old *A. arborescens* plants for use in Alzheimer’s disease. They noticed the ability of the extracts to inhibit radical production with the consequent protection from toxicity induced by beta-amyloid, which is another mechanism for the development of Alzheimer’s disease (the beta-amyloid hypothesis) [53,54]. Therefore, it can be suspected that the neuroprotective effect of *A. arborescens* in Alzheimer’s disease will be multidirectional. 

The literature on the subject presents the benefits of using various species of *Aloe* in skin problems [55,56]. Wound healing potential was proven for its fresh form in many species of the genus *Aloe*, similar to lyophilized forms. The healing properties of fresh *Aloe vera* gel have been confirmed in studies in cases of, e.g., burn wounds, diabetic ulcers, or nipple soreness [57,58,59]. During the scratch test, we confirmed that the extract of lyophilized *A. arborescens* gel facilitates the in vitro healing process compared to the control (without using any healing facilitators, untreated), which was found earlier for the lyophilized gel form of selected species of *Aloe* by Fox et al. [60]. Therefore, it can be suspected that although the freeze-drying process, like other processing methods, may cause some changes in the composition of the raw material—which was confirmed for *Aloe vera*—in the studied case, it did not result in the loss of healing properties [61,62]. However, comparing fresh gel and freeze-dried gel seems to be an issue worth investigating. 

Other authors have also studied the antimicrobial properties of aloes [22]. Kupnik et al. (2021) conducted a comparative study in which they tested natural aloe samples (juice and gel) and commercial products derived from aloe vera. The authors indicated aloe-based products had antimicrobial and bacteriostatic activity against all tested microorganisms. Studies have shown that *A. arborescens*’ natural juices and gels are in higher added concentrations than commercial aloe products, especially against *Bacillus cereus, Candida albicans,* and *Pseudomonas aeruginosa* cultures whose growth has been completely inhibited. These authors also demonstrated the antimicrobial properties of aloe vera juice against *S. aureus* bacteria. Both the results obtained in this study and the work of Kupnik et al. (2021) indicate the great potential of *A. arborescens* for further use in the medical, cosmetic, food, and pharmaceutical industries as a raw material with high functional potential, but also for to its antimicrobial properties as a natural source of preservative compounds. It is also worth pointing out that very few studies in the scientific literature have shown the antimicrobial effect of *A. arborescens* in commercial products and fresh juice or extract on microorganisms.

## 4. Materials and Methods

### 4.1. Plant Material

Fresh leaves of 3-year-old *Aloe arborescens* were provided by Phytopharm Klęka S.A. (Poland) from plants growing in controlled, inbreed plantations in heated greenhouses. Plants were provided controlled temperature hydration and fertilization conditions (Supplementary Materials Figure S1–S3). 

The cultivation was carried out, depending on the season, at 10–30 °C, regulated with shading and ventilation, which, apart from the temperature control, also allowed for humidity modification. The temperature in the greenhouse did not drop below 0 °C because plants do not tolerate such temperatures. The water used for watering the plantation was laboratory-controlled. Fertilizers were minimized—organic and mineral fertilizers were used only in necessary amounts. The risk of contamination was minimal due to the monoculture type of plantation and the lack of pesticides, fungicides, herbicides, and fumigants.

Propagation was carried out vegetatively by shoots, which were first transferred to pots and then, after about a year, placed in the ground. The age of the plantation was counted from the moment the seedling was planted in the ground. Fresh leaves were harvested by hand by cutting the entire plant.

### 4.2. Lyophilisation of Aloe Gel

The gel from the leaves of 3-year-old *A. arborescens* plants was obtained by cutting off leaf edges and separating the inner part of the leaves from the peel. Obtained pulp was placed in round bottom flasks and frozen at –20 °C. The flasks with frozen pulp were connected to freeze-dryer manifolds (HetoPowerDry PL3000 Freeze Dryer (Thermo Scientific, Schwerte, Germany). The lyophilization process lasted for 72 h at the temperature of –55 °C under vacuum conditions. Lyophilised pulp was protected against humidity and light.

### 4.3. Extracts Preparation

The lyophilised gel from the 3-year-old *A. arborescens* plants prepared the extract intended for further studies. For this purpose, the following procedure was applied: 1000 µg of the lyophilised milled gel was extracted with ethanol 96% (*v/v*) for 30 min at 30 °C with the use of ultrasounds. The extraction process was performed three times under the given conditions. After completing each step, the supernatant was centrifuged and decanted. The collected supernatants were mixed, filtered through the syringe filter with a pore diameter of 0.45 µm, then concentrated to dryness by applying rotary evaporation (Buchi Rotavapor R-210). The residues were dissolved in 1 mL of ethanol 96% (*v/v*). The extract prepared in this way was used to perform biological activity tests.

### 4.4. HPLC-DAD Method

The HPLC-DAD method was applied to confirm the identity of the material. For this purpose, two standard substances were used as standards—aloin A and aloenin A. The HPLC Dionex (Thermoline Fisher Scientific, Schwerte, Germany) was equipped with a DAD detector. The software was Chromeleon software version 7.0 from DionexThermoline Fisher Scientific (Waltham, MA, US). The stationary phase was LiChrospher 100 RP-18e (250 mm × 4.6 mm, 5 µm) HPLC column (Merck, Warsaw, Poland). Gradient flows of the A-methanol and B-water phases were used at 1 mL/min. Then, 20 µL of the sample was injected onto the column, and the detection was performed at the wavelength λ_max_ = 295 nm.

### 4.5. The Sum of Polyphenolic Compounds

The method was based on a color reaction of the Folin–Ciocâlteu reagent with the contained phenolic groups in sodium carbonate. Therefore, 1 mL of the extract was vortexed with 1 mL of Folin–Ciocâlteu reagent for 3 s, then 1 mL of sodium carbonate and 7 mL of water were added and remixed in the vortex for 3 s. After 90 min of incubation in the dark, the resulting colored complex was determined spectrophotometrically at a wavelength of 765 nm in the Metertech SP-830 spectrophotometer (Metertech, Taipei, Taiwan). In order to determine the content of compounds reactive with the Folin–Ciocâlteu reagent, a standard curve for gallic acid was established. Then, using the determined curve, it was converted to the content in 1 g of the dry mass of the extract.

### 4.6. The Sum of Phenolic Acids

The presence of phenolic acid compounds was determined by the colorimetric method described in Polish Pharmacopoeia VI. The study was performed by adding to 5 mL of distilled water in a volumetric flask, analyzed extract, 0.5 M solution of hydrochloric acid, Arnov’s reagent, and sodium hydroxide in a volume of 1 mL each. After diluting with distilled water to 10 mL volume, spectroscopy measurement was performed at the wavelength of 490 nm.

The content of phenolic acids was converted to caffeic acid based on the drawn standard curve and expressed in mg per 1 g of dry mass.

### 4.7. Comet Assay

The genotoxic potential of *Aloe arborescens* extracts was evaluated using a single-cell gel electrophoresis technique based on OxiSelect Comet Assay Kit (Cell Biolabs, Inc., San Diego, CA, USA). All the procedures performed were in line with the manufacturer’s protocol. Human Hs27 cells, previously exposed for 24 h to the *Aloe vera* extract, were combined with liquefied low melting agarose at 37 °C and transferred onto the OxiSelect comet slides. After storing for 15 min at 4 °C, the slides were immersed in a pre-chilled Lysis Buffer (45 min, 4 °C) and Alkaline Solution (for 30 min at 4 °C). Afterward, the Alkaline Solution was replaced with a pre-chilled TBE (Tris/borate/EDTA) electrophoresis solution. After immersing for 5 min, the slides were put into the horizontal electrophoresis chamber and covered with TBE electrophoresis buffer. The electrophoresis was run for 15 min at 1 V/cm. Finally, DNA was stained with Vista Green DNA Dye for 15 min at room temperature and observed under a fluorescence microscope (Olympus BX63). The images of cells were captured using XM10 digital camera (Olympus). The DNA damage was measured quantitatively using ImageJ software (NIH, Bethesda, MD, USA). Etoposide was used as the positive control. At least 50 randomly selected images were used in each analysis.

### 4.8. Enzyme Inhibition Assays

#### 4.8.1. Hyaluronidase Inhibition Assay

The assay of hyaluronidase activity inhibition was performed according to the modified turbidimetric method of Grabowska et al. [63]. The principle of the method is to spectrophotometrically measure the turbidance resulting from the presence of precipitated hyaluronic acid remaining in the reaction mixture. High turbidity indicates a potent inhibition of the acid-degrading hyaluronidase activity by the tested extract—the greater the turbidity, the more the enzyme is inhibited. The test was carried out using a 96-well plate. The tested sample (TS) and five blank samples (B1–B5) were prepared in six repetitions. To prepare the tested sample, 25 μL of a 0.5% solution of bovine serum albumin in acetate buffer of pH 4.5 (incubation buffer), 25 μL of hyaluronidase solution, 10 μL of the extract, and 15 μL of acetate buffer (pH = 4.5) were mixed in wells and incubated at 37 °C for 15 min. When the time passed, 25 μL of hyaluronic acid (0.3 mg/mL) was added, and the plate was incubated at 37 °C for 45 min. Finally, 200 μL of CTAB was added, and after 10 min of incubation at room temperature, spectrophotometric measurement was performed at the wavelength of 600 nm. Blank samples were prepared analogically. In blank 1, the hyaluronidase and hyaluronic acid solutions were replaced with the acetate buffer. Blank 2 also did not contain hyaluronidase, and the tested extract was replaced with the medium used to prepare it. Blank 3 likewise replaced the sample with the pure medium. Blank 4 did not contain hyaluronic acid, and blank 5 did not contain hyaluronidase.

The percentage of inhibition of hyaluronidase activity was determined according to the following equation:

$$I\%\;=\frac{\left(A_{S}-A_{B4}\right)-\left(A_{B3}-A_{B1}\right)}{\left(A_{B5}-A_{B4}\right)-\left(A_{B3}-A_{B1}\right)}\;*\;100\%$$

where I% is the % inhibition of hyaluronidase; A_S_—absorbance of the sample; A_B1_—absorbance of blank 1; A_B3_—absorbance of blank 3; A_B4_—absorbance of blank 4; A_B5_—absorbance of blank 5.

The kaempferol solution was prepared as a standard, and it was handled as the test sample, conducting the test according to the scheme and calculating the percentage of inhibition of hyaluronidase activity.

#### 4.8.2. α-Glucosidase Inhibition Assay

The assay was carried out according to the modified Chipiti et al. [64] method with modifications. The test is based on measuring the change of absorbance after inducing a reaction in which, under the influence of α-glucosidase, yellow p-nitrophenol is formed from pNPG. The spectrophotometric measurement is performed at a wavelength of λ = 405 nm. The more potent the α-glucosidase inhibitor in the tested substance is, the less yellow the solution will be, as minor p-nitrophenol will be released during the reaction of pNPG with the enzyme.

In order to perform the assay, 50 μL of 0.1 M phosphate buffer (pH = 6.8), 50 μL of the sample, and 30 μL of α-glucosidase solution (0.5 U/mL) were dispensed into wells of the plate. Blank samples were prepared for self-control. The blank for the test solution was a sample with the volume of the enzyme replaced with a buffer. The pure medium used to prepare the extract was added to the control sample instead of the extract. In the blank for the control sample, the medium was used instead of the extract, and the enzyme was replaced with a buffer. All tested and blank samples were repeated six times. The plates protected against light access were shaken for 5 min and then incubated at 37 °C for 10 min. Then, 20 µL of pNPG was added to each well and incubated for 20 min at 37 °C. After this time, 100 μL of sodium carbonate was added to each sample, followed by shaking for 2 min, also at 37 °C. Absorbance was measured at a wavelength of λ = 405 nm. The result was calculated using the formula:

$$\mathrm{enzyme}\;\mathrm{inhibition}\;\left(\%\right)=\left(\frac{A_{C}-A_{S}}{A_{C}}\right)\times 100\%$$

where A_C_ is the absorbance of the control sample, and A_S_ is the absorbance of the sample.

#### 4.8.3. Acetylcholinesterase and Butyrylcholinesterase Inhibition Assay

The extract was evaluated for inhibiting activity by acetylcholinesterase (AChE) and butyrylcholinesterase (BChE). The test was carried out according to the spectrophotometric method developed by Kobus-Cisowska et al. [65]. The assay was performed by mixing 5 µL of the sample, 60 µL of 0.05 M Tris-HCl buffer of pH = 8, 30 µL of AChE or BChE, and incubating the plate at 25 °C for 5 min. Then, 30 µL of acetylthiocholine iodide (ATCI) or butyrythiocholine iodide (BTCI) and 125 µL of DTNB were added, and the plate was incubated at 25 °C. The color change caused by the hydrolysis of acetylthiocholine iodide and butyrythiocholine iodide by cholinesterase enzymes was measured at the wavelength λ = 405 nm after 30 min for AChE, and after 20 min for BChE. Eserine was used as a reference cholinesterase inhibitor. In the blank for the test samples, the enzyme solution was replaced with a buffer; in control, the medium used for the extraction was used instead of the test solution, and in the blank for the control, in addition to changing the extract to a pure medium, the enzyme was replaced with an appropriate volume of the buffer. The inhibition potential was calculated according to the equation:

$$\mathrm{enzyme}\;\mathrm{inhibition}\;\left(\%\right)=100-\frac{(A_{S}-A_{\mathrm{SB}})\times 100}{A_{C}-A_{\mathrm{CB}}}$$

where A_S_ is the absorbance of the sample; A_SB_—absorbance of the sample’s blank; A_C_—absorbance of the control; A_SB_—absorbance of the control’s blank.

The results were presented as the eserine equivalents.

### 4.9. Scratch Assay 

A wound-healing assay was conducted using normal human skin fibroblasts (CRL-1474 ATCC). The preparation stage for the study included culturing the fibroblasts in a humified environment at 5% carbon dioxide at a temperature of 37 °C, then collecting them from subconfluent monolayers with trypsin/EDTA. The scratch test that measures the ability to heal wounds was performed according to the following procedure. The 6-well plate was used to seed the cells (1 × 10^5^ cells/mL), which were later cultivated in Dulbecco’s modified Eagle medium (DMEM), high glucose supplemented with 10% fetal bovine serum (FBS), penicillin (100 U/mL), and streptomycin (100 µg/mL). After reaching about 90% of cell confluence, the vertical linear scratch-simulating wound was made in the monolayer using a sterile pipette tip, then washed three times with phosphate buffer saline (PBS) to remove cellular debris. The medium was then applied with the prepared concentrations of *A. arborescens* leaf gel lyophilisate (100 µg/mL and 500 µg/mL). The control samples were wells with 2% PBS. Three repetitions were made for each trial.

The microscopic photos of the scratch were taken every 12 h, starting from the 0 time point (0 h—right after creating the scratch) to 36 h using the Olympus CKX53 device equipped with a digital camera. Measuring the open wound area was possible thanks to ImageJ software, and the obtained values allowed for the calculation of wound closure according to the formula:

$$\mathrm{wound}\;\mathrm{closure}\;\left(\%\right)=\frac{\mathrm{open}\;\mathrm{wound}\;\mathrm{area}\;\left(\mathrm{at}\;0\;h\right)-\mathrm{open}\;\mathrm{wound}\;\mathrm{area}\;\left(\mathrm{at}\;12/24/36\;h\right)}{\mathrm{open}\;\mathrm{wound}\;\mathrm{area}\;\left(\mathrm{at}\;0\;h\right)}\;*\;100$$

### 4.10. Antimicrobial Studies

The antimicrobial activity of the studied extract of lyophilized *Aloe arborescens* gel was tested by applying the agar well diffusion method. The reference strains cultured in thioglycolate broth were inoculated on a blood agar plate. Then, 100 µL of the studied extract (1 g dry weight of freeze-dried aloe pulp per 1 mL of 96% ethanol) was added to wells (5 mm × 5 mm) prepared on the agar plate’s surface; moreover, positive and negative controls were performed using 5.25% sodium hypochlorite and 0.9% sodium chloride, respectively. The plates were incubated for 24 h at 37 °C. The diameter of the clear zone (growth inhibition zone) around the wells with the extracts measured the antimicrobial activity.

The following microorganisms were used for the study: Gram-positive bacteria: *Clostridium difficile* ATCC 9689, *Clostridiumbutyricum* ATTC 860, *Listeriamonocytogenes* ATCC 7644, *Bacillussubtilis* ATCC 238557, *Enterococcusfaecalis* ATTC 29212, *Staphylococcusaureus* ATCC 25923, *Staphylococcuspyogenes* ATCC 19615; Gram-negative bacteria: *Escherichia coli* ATCC 25922, *Klebsiella pneumonia* ATCC 31488, *Proteus mirabilis* ATCC 12453, *Salmonella typhimurium* ATCC 14028, *Pseudomonas aeruginosa* ATCC 27853, *Enterobacter aerogenes* ATCC 13048; yeast: *Candida krusei* ATCC 14243, *Candida albicans* ATTC 10231.

## 5. Conclusions

In summary, the performed studies confirm the multi-level activity of *Aloe arborescens*. The test performed on Hs27 fibroblasts confirmed the lack of genotoxic effects of the investigated aloe extract. The tested extracts did not stimulate genotoxicity; thus, the safety of using the raw material was confirmed. The following research stage determined the potential action profile by determining the enzyme inhibition capacity. The determination of the inhibition of hyaluronidase was performed as a determinant of the anti-inflammatory capacity. The tested extract showed higher activity than kaempferol, a control substance with confirmed activity against the enzyme. To confirm the hypoglycemic activity, a α-glucosidase inhibition activity assay was used to prove superior plant extract activity compared to acarbose, used as a control substance. As a marker of neuroprotective activity, an acetylcholinesterase and butyrylcholinesterase inhibition study was performed. In addition, a scratch test was carried out to confirm the healing abilities of the skin application; the obtained results indicated a positive effect of even a highly diluted extract. The further study of microbial activity by examining the bright spot zones showed a broad spectrum of activity against Gram-positive and Gram-negative bacteria and yeasts. 

Due to the obtained results, it is possible to conclude the safe and effective use of extracts from the tested material in application to the skin to improve wound healing and protect them against potential infection with pathogenic microorganisms. In addition, the obtained results indicate the potential of using *Aloe arborescens* as an effective inhibitor of a-glucosidase in supporting the therapy of type 2 diabetes. The multi-level effect of the raw material has been additionally confirmed by demonstrating that the inhibition of AChE and BChE translates into the nervous system’s response can be used, for example, in the treatment of Alzheimer’s disease.

The obtained results confirm the multi-level action of *Aloe arborescens* and a wide spectrum of potential application; the product can be used for a broad spectrum of diseases with diverse routes of administration.

## Figures and Tables

**Table 1:** The content of active compounds in the tested freeze-dried *Aloe* leaf.

Compound	Content in a Freeze-Dried Leaf (mg/g d.m.)
Aloin A	1.44 ± 0.24
Aloenin A	3.98 ± 0.89
Sum of polyphenols	352.24 ± 1.75
Sum of phenolic acids	187.38 ± 12.88

**Table 2:** Diameters of inhibition zones for selected microorganisms.

Microorganism	Diameter of Inhibition Zone (mm)—*Aloe arborescens* Extract	Diameter of Inhibition Zone (mm)—5.25% Sodium Hypochlorite
*Clostridium difficile* ATCC 9689	0.8	15.3
*Clostridium butyricum* ATTC 860	1.3	12.1
*Listeria monocytogenes* ATCC 7644	4.0	18.4
*Bacillus subtilis* ATCC 238557	8.2	16.8
*Enterococcus faecalis* ATTC 29212	2.2	23.1
*Staphylococcus aureus* ATCC 25923	9.1	18.9
*Staphylococcus pyogenes* ATCC 19615	8.9	21.3
*Escherichia coli* ATCC 25922	9.2	18.9
*Klebsiell apneumoniae* ATCC 31488	11.3	24.5
*Proteus mirabilis* ATCC 12453	4.3	19.8
*Salmonella typhimurium* ATCC 14028	3.8	17.7
*Pseudomonas aereginosa* ATCC 27853	9.3	24.8
*Enterobacter aerogenes* ATCC 13048	1.2	19.3
*Candida krusei* ATCC 14243	9.2	18.3
*Candida albicans* ATTC 10231	10.7	20.1

## Data Availability

Data from analysis are available from the authors.

## Supplementary Materials

The following supporting information can be downloaded at: https://www.mdpi.com/article/10.3390/molecules27072323/s1, Figure S1. Cultivation of *Aloe arborescens* in the 3rd year of growth; Figure S2. A method of obtaining leaves from mature plants of *Aloe arborescens;* Figure S3. Freshly cut leaf of *Aloe arborescens* prepared to obtain the pulp. Figure S4. *Aloe arborescens* leaf: freshly cut (A) and cross-section with visible pulp (B).

## Sample Availability

Samples of the plant raw are available from the authors.
