# Supplementary material for: Aloe arborescens: In Vitro Screening of Genotoxicity, Effective Inhibition of Enzyme Characteristics for Disease Etiology, and Microbiological Activity
Source: Molecules. 2022 Apr 3;27(7):2323. doi: 10.3390/molecules27072323 (PMC9000289; doi:10.3390/molecules27072323)
Supplement: Supplementary file 1 [file molecules-27-02323-s001.zip › molecules-1637100-supplementary.pdf]

molecules

MDPI

Article

Aloe arborescens–In Vitro Screening of Safety, Effective Inhibition of Enzymes Characteristic for Disease Etiology and Microbiological Activity

Kamil Pawłowicz [1,2], Szymon Sip [2], Tomasz Plech [3], Barbara Kaproń [4], Joanna Kobus-Cisowska [5], Judyta Cielecka-Piontek[2,*]

[1] Phytopharm Klęka S.A., Klęka 1, 63-040 Nowe Miasto nad Warta, Poland; Kamil.pwałowicz@europlant-group.pl (K.P.)
[2] Department of Pharmacognosy, Poznan University of Medical Sciences, Rokietnicka 3, 60-806 Poznań, Poland; dsiakowska@ump.edu.pl (D.L.),szymonsip@ump.edu.pl (S.S.), jpiontek@ump.edu.pl (J.C.-P.)
[3] Department of Pharmacology, Medical University of Lublin, Chodźki 4a, 20093 Lublin, Poland; tomasz.plech@umlub.pl (T.P.)
[4] Department of Clinical Genetics, Medical University of Lublin, Radziwiłłowska 11, 20-080 Lublin, Poland; barbara.kapron@umlub.pl (B.K.)
[5] Department of Gastronomy Sciences and Functional Foods, Faculty of Food Science and Nutrition, Poznan University of Life Sciences (PULS), Wojska Polskiego Str. 28, 60-637 Poznań, Poland; joanna.kobus-cisowska@up.poznan.pl (J.K.-C.)
* Correspondence: : jpiontek@ump.edu.pl

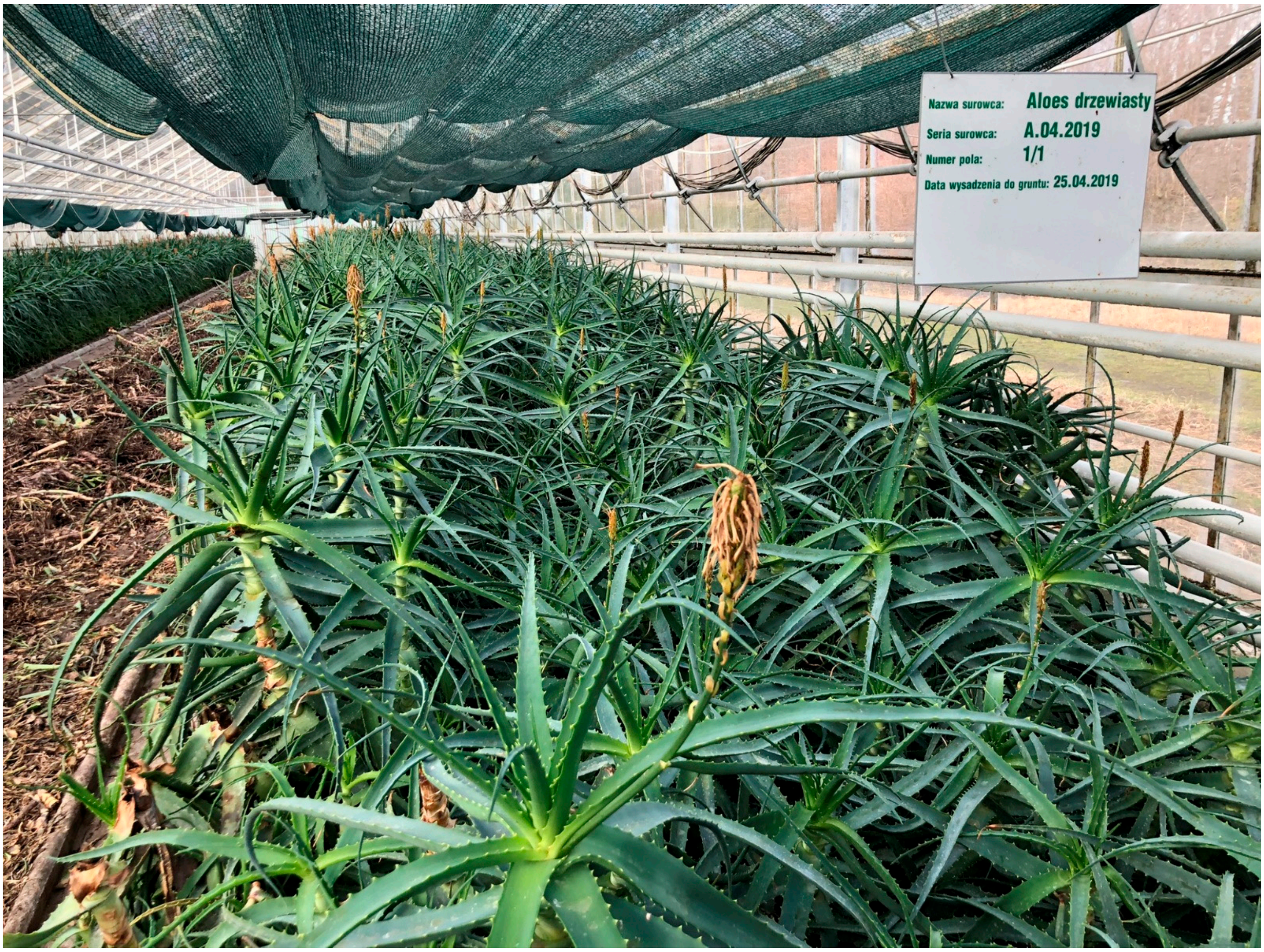

Figure S1. Cultivation of *Aloe arborescens* in the 3rd year of growth.

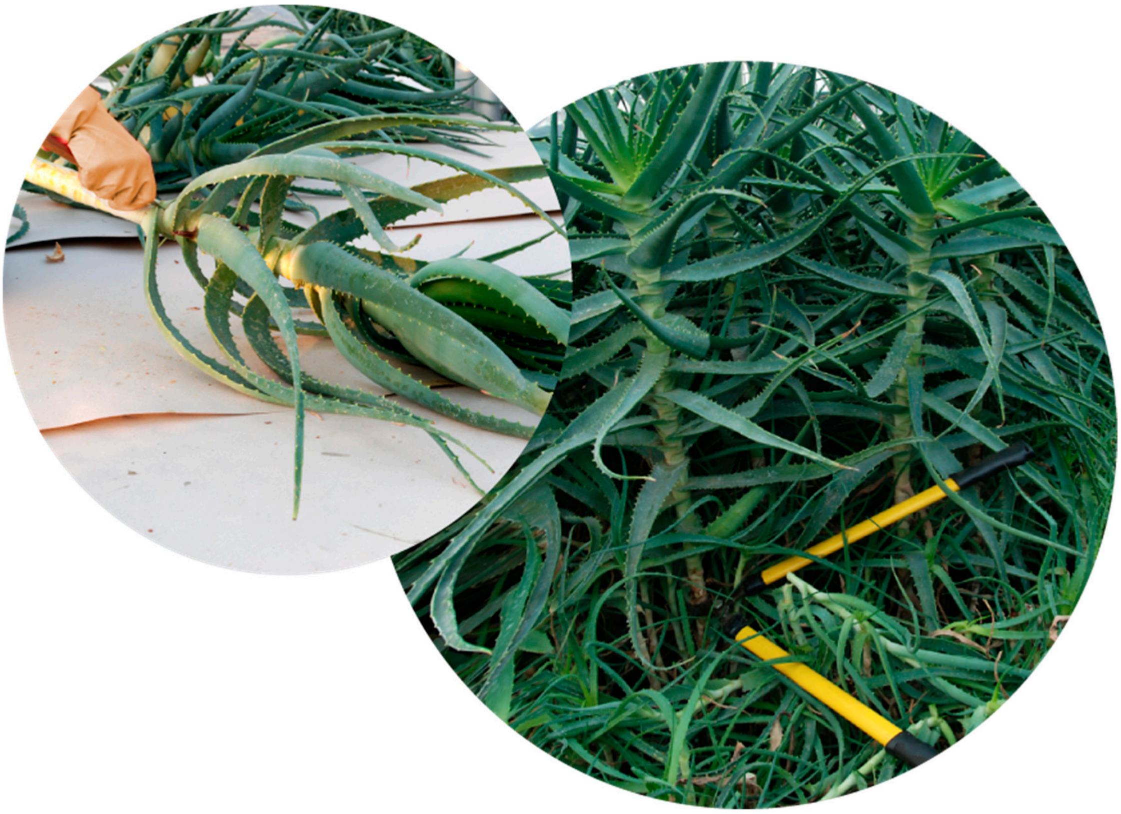

Figure S2. A method of obtaining leaves from mature plants of *Aloe arborescens*.

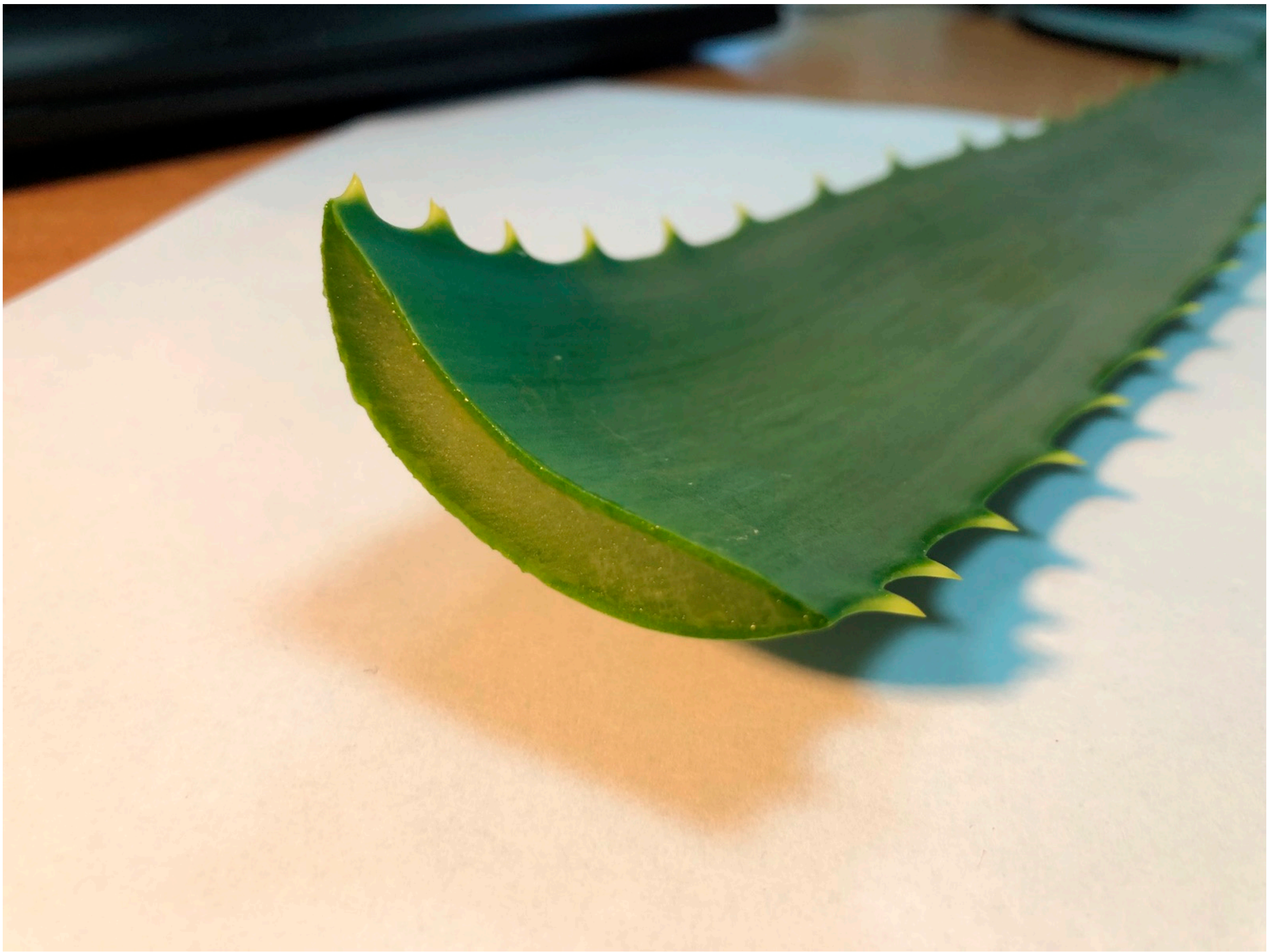

Figure S3. Freshly cut leaf of *Aloe arborescens* prepared to obtain the pulp.

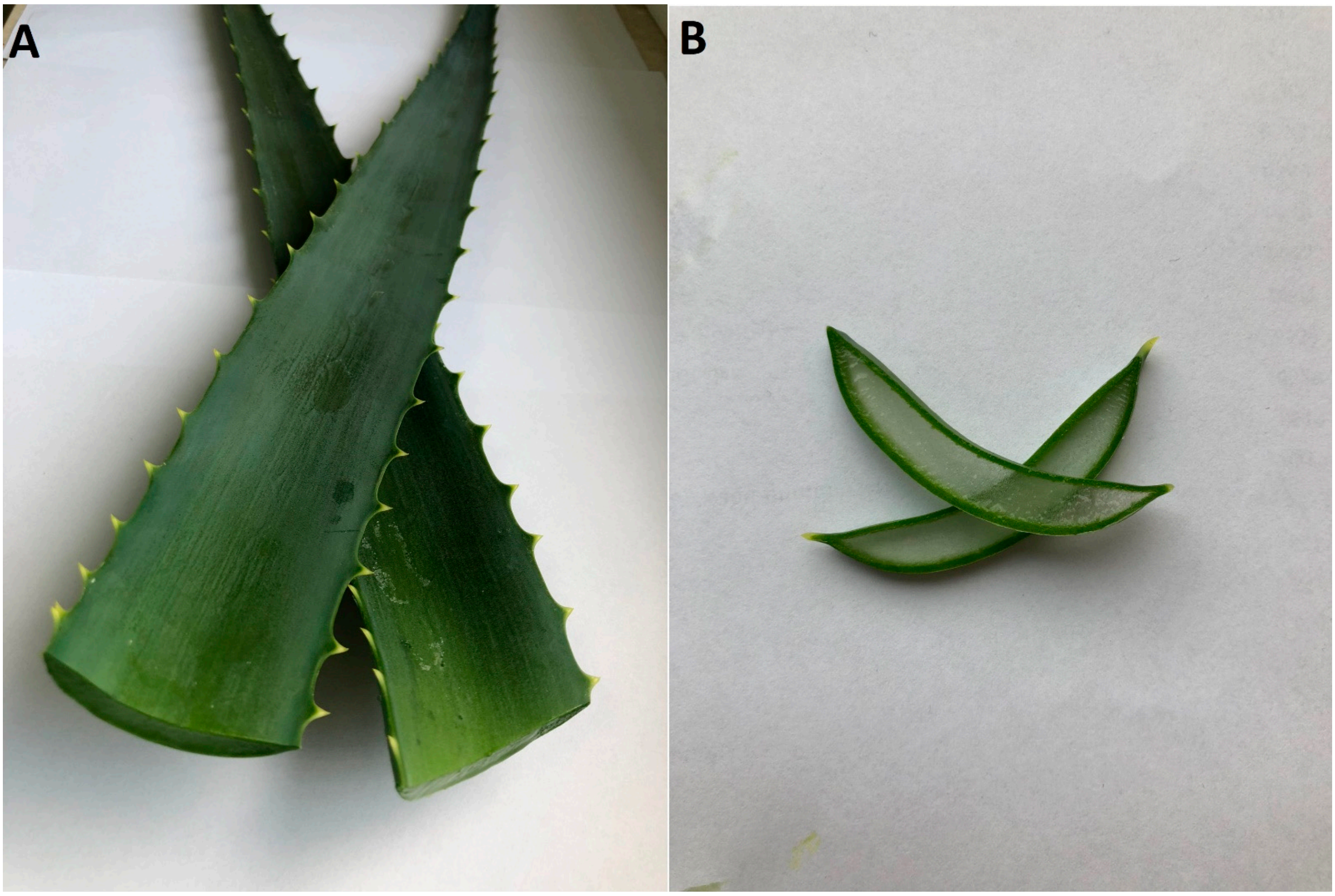

Figure S4. *Aloe arborescens* leaf : freshly cut (A) and cross-section with visible pulp (B).
